# Silencing of let-7b-5p inhibits ovarian cancer cell proliferation and stemness characteristics by Asp-Glu-Ala-Asp-box helicase 19A

**DOI:** 10.1080/21655979.2021.1982276

**Published:** 2021-10-06

**Authors:** Xiujuan Huang, Hongxia Dong, Yang Liu, Fen Yu, Shunshi Yang, Zhen Chen, Jueying Li

**Affiliations:** a Department of Ultrasound, The Central Hospital of Wuhan, Tongji Medical College, Huazhong University of Science and Technology, Wuhan Hubei, China; b Department of Emergency, The Central Hospital of Wuhan, Tongji Medical College, Huazhong University of Science and Technology, Wuhan Hubei, China

**Keywords:** OVCA, let-7b-5p, stemness characteristics, ddx19a, mb

## Abstract

The emergence and recurrence of ovarian cancer are associated with ovarian cancer stem cells. For cancer treatment, gene delivery of microbubbles (MB)-mediated microRNA (miRNA) is considered as a promising approach. In this study, our aim is to investigate the effects of MB-mediated let-7b-5p inhibitor on the proliferation and stemness characteristics of ovarian cancer (OVCA) cells. Let-7b-5p inhibitor mediated by MB was prepared (termed MB-let-7b-5p inhibitor), and the effects of MB-let-7b-5p inhibitor and let-7b-5p inhibitor on OVCA cell viability, proliferation and stemness characteristics were investigated. We found that MB-let-7b-5p inhibitor presented a higher transfection efficiency than let-7b-5p inhibitor alone. The inhibitory effect of MB-let-7b-5p inhibitor on OVCA cells was more significant than let-7b-5p inhibitor. Let-7b-5p targeted DEAD (Asp-Glu-Ala-Asp)-box helicase 19A (DDX19A), which was downregulated in OVCA cells. The downregulation of DDX19A reversed the inhibitory effects of MB-let-7b-5p inhibitor on OVCA cells. To sum up, we found that MB-let-7b-5p suppressed OVCA cell malignant behaviors by targeting DDX19A.

## Introduction

Ovarian cancer (OVCA), associated with high morbidity, is the main cause of cancer-related death in the female reproductive system [1]. OVCA is featured with an initial asymptomatic period and aggressive growth [2]. Although the treatments have been improved a lot over the past years, lack of effective detection methods and evident symptoms for the early stage of OVCA leads to the tragedy that 75% of patients are diagnosed at the advanced stage [3]. Approximately 70% of the patients with advanced OVCA (stage III or IV) have a poor prognosis after traditional therapies (mainly including chemotherapy, radiotherapy, and surgery), and the five-year survival rate of OVCA patients is less than 50% [4]. It was reported that there were approximately 313,959 new OVCA and 207,252 new OVCA deaths cases in 185 countries, taking up 1.6% and 2.1% of all new cancer cases and new cancer deaths, respectively, in 2020 [5]. The poor prognosis and low five-year survival rate are due to the high recurrence and chemotherapy resistance of ovarian cancer stem cells (OCSCs). Therefore, novel and effective treatment targeting OCSCs is in urgent need. Cancer stem cells (CSCs) are stem cell-like cells with resistance to radiotherapy, high tumorigenicity, non-directional differentiation, replication, self-renewal ability [6,7]. With these characteristics that promote and maintain tumor growth, CSCs are considered to be the cause of cancer occurrence, recurrence, metastasis and resistance [8,9].

MicroRNAs (miRNAs) play an important role in the epithelial-to-mesenchymal transition (EMT) and proliferation of cancer cells by modulating the expression of mRNAs [10]. Accumulating evidence has revealed that miRNAs and mRNAs can act as novel diagnostic or therapeutic targets for OVCA treatment [11,12]. Take miR-802 as an example, it suppresses the growth, migration and induces the apoptosis of epithelial OVCA cells by targeting mRNA tyrosine 3-monooxygenase/tryptophan 5-monooxygenase activation protein zeta [13]. MiR-424-5p is a tumor suppressor in OVCA and it takes effects via targeting acyl-CoA synthetase long-chain family member 4 [14]. The miRNA let-7b-5p has been studied in prostate cancer [15], glioma [16], hepatocellular carcinoma [17]. Moreover, an exploration made by Liu J *et al*. mentioned that let-7b-5p is upregulated in ovarian cancer [18]. Contradictively, some authors proposed a different view that let-7b-5p is downregulated and plays an antioncogenic role in OVCA [19–21]. We detected the expression of let-7b-5p in OVCA and identified the upregulation of let-7b-5p in OVCA tissues and cells.

Recently, microbubbles (MBs) are considered as ultrasound-assisted gene delivery tools to accelerate gene entering cells and disturbing cell membranes [22]. The short-term membrane permeability of surrounding cells is caused by MBs under ultrasonic irradiation, facilitating targeted local administration without damaging the cells [23]. Nowadays, a combination of ultrasound and microbubbles (USMB) is employed for noninvasive enhancement of uptake of genes and drugs [24]. MBs promote ultrasound-mediated gene transfer efficiency in cell culture and selectively transfer therapeutic genes to disease sites [25]. USMB-mediated gene delivery has been recognized as an effective tool for the treatment of malignant tumors [26]. For example, USMB-mediated miR-378 was found to inhibit proliferation and induce apoptosis of hepatoma cells [27]. Wu J *et al*. suggested that USMB-mediated silencing of enhancer of zeste 2 polycomb repressive complex 2 subunit inhibits liver cancer stem cell stemness [28]. Intriguingly, USMB enhanced transfection efficiency of let-7b-5p mimics, and USMB-mediated transfection has a better suppressive effect on the apoptosis of CD133^+^ OCSCs [29].

Considering the upregulation of let-7b-5p in OVCA tissues and cells, we hypothesized that let-7b-5p exerts an oncogenic role in OVCA. The present study was designed to compare the effects of USMB-mediated let-7b-5p inhibitor and the effects of let-7b-5p inhibitor alone on the proliferation and stemness characteristics of OVCA cells, and subsequently explored the downstream target of let-7b-5p.

## Materials and methods

### Tissue samples and cell lines

Twenty-eight paired adjacent nontumor tissues and OVCA tissues were obtained from patients with OVCA at the Central Hospital of Wuhan, Tongji Medical College, Huazhong University of Science and Technology (Hubei, China). The patients underwent surgical resection. No preoperative radiotherapy or chemotherapy was given to the patients. Characteristics of the 28 patients are provided in Table 1. After dissection, tissues were immediately frozen in liquid nitrogen, and subsequently stored at −80°C until use for further analysis. The written informed consents were signed by all the participants in this study, and this study was approved by the Ethics Committee of the Central Hospital of Wuhan, Tongji Medical College, Huazhong University of Science and Technology (Hubei, China). These archive samples were used for expression analysis of let-7b-5p and its target genes.

**Table 1.** Characteristics of patients with ovarian cancers

Characteristics	Number (N = 28)
Age	Median: 42 years Range: 21–70 years
FIGO stage	
I	5 (17.9%)
II	8 (28.6%)
III	12 (42.8%)
IV	3 (10.7%)
ECOG	
0	2 (7.1%)
I	10 (35.7%)
II	9 (32.1%)
III	7 (25%)
Histologic subtypes	
Serous	21 (75%)
Endometriods	6 (21.4%)
Clear cell	1 (3.6%)

Human ovarian surface epithelial cell line (HOSEpiC) and the human OVCA cell lines including SKOV3 and CAOV3 cells were purchased from the American Type Culture Collection (ATCC; Manassas, VA, USA). All cells were cultured in Dulbecco’s modified Eagle’s medium (DMEM) together with 10% fetal bovine serum (FBS; Invitrogen, MA, USA). The cell lines were incubated in a humidified atmosphere at 37°C containing 5% CO_2_.

## Cell transfection

GenePharma (Shanghai, China) provided the let-7b-5p inhibitor (5ʹAACCACACAACCUACUACCUCA-3ʹ) and short hairpin RNAs (shRNAs) targeting DEAD (Asp-Glu-Ala-Asp)-box helicase 19A (DDX19A) (sh-DDX19A: 5ʹ-GCTGTCAAGTCGATGACCAATTTCAAGAGAATTGGTCATCGACTTGACAGCTTTTTT-3ʹ) as well as their negative controls (NC inhibitor: 5ʹ-GUUGAUUAUGGUGGUGUGAGUG-3ʹ and sh-NC: 5ʹ-ACTGTAACCGGATCGCAGTATTTCAAGAGAATACTGCGATCCGGTTACAGTTTTTTT-3ʹ). Lipofectamine 3000 (Invitrogen) was employed for cell transfection following the recommendations of the manufacturer. After transfection for 48 h, reverse transcription quantitative polymerase chain reaction (RT-qPCR) was applied to detect the transfection efficiency. Transfected cells were employed for further experiments.

## Microbubble preparation

The preparation of MB-let-7b-5p was as follows: first, MBs were synthesized by ultrasonic dispersion of 0.4 mg/mL 1,2-bisstearoyl-3-trifluoromethypropane, 2 mg/mL 1-bisstearoyl phosphatidylcholine, 1 mg/mL polyethylene glycol-40 stearate, and decafluorobutane (Avanti Polar Lipids Inc., Alabaster, AL, USA) in a water box. Next, an inverted fluorescence microscope (DM 4000B, Leica, Germany) was employed to observe the MBs. The size of MBs was detected by a nanometer particle size analyzer (NS-90, OMEC Instruments Co., Ltd., Guangdong, China). The average particle size was about 2.16–4.68 μm. A 1 μm filtration membrane was utilized to filter the MBs, which was adjusted into 0.8–1.6 × 10^9^/mL. After that, 1 μg let-7b-5p inhibitor was blended with 50 μL MB suspension and cultured for 30 minutes at 37°C. To harvest MB-let-7b-5p, 0.16 M phosphate buffer saline (PBS) was used to remove the unbounded let-7b-5p. The harvested mixture was added into SKOV3 and CAOV3 cells for transfection using Lipofectamine 3000 (Invitrogen) under the manufacturer’s guidelines.

## RT-qPCR

RNA expression in OVCA cells and tissues was assessed by RT-qPCR analysis. First, total RNAs were extracted from OVCA cells and tissues using TRIzol reagent (Takara Biotechnology, Dalian, China) under manufacturer’s instructions. Reverse transcription of 1 µg total RNA into cDNA was performed by a First Strand cDNA Synthesis kit (Takara Biotechnology). The SYBR Green Master Mix (Takara Biotechnology) was employed to detect the RNA expression, with glyceraldehyde-3-phosphate dehydrogenase (GAPDH) or U6 as an internal control. Relative expression of RNAs was calculated according to the 2^−*ΔΔ*Ct^ method [30]. In this assay, we used the following primers:

Let-7b-5p: forward 5ʹ-GCGCTGAGGTAGTAGGTTGTG-3ʹ

Reverse 5ʹ-GTGCAGGGTCCGAGGT-3ʹ

DDX19A: forward 5ʹ-AAAGTGATTGAGCAGATGGG-3ʹ

Reverse: 5ʹ-GCCTCTTTCCAATTTATTGCC-3ʹ

GAPDH: forward 5ʹ-TCATTTCCTGGTATGACAACGA-3ʹ

Reverse 5ʹ-GTCTTACTCCTTGGAGGCC-3ʹ

U6: forward 5ʹ-TGCTATCACTTCAGCAGCA-3ʹ

Reverse 5ʹ-GAGGTCATGCTAATCTTCTCTG-3ʹ

## Cell viability assay

Cell viability was evaluated by a Cell Counting Kit-8 (CCK-8; Dojindo, Kyushu, Japan). First, cells were inoculated into a 96-well plate at a density of 1 × 10^3^ cells/mL and pre-cultured for 24 h. Second, after incubation for 48 h, 10 μL of CCK-8 reagent (HY-K0301, MedChemExpress, NJ, USA) was added to each well. Third, the cells were incubated with CCK-8 reagent for 2–4 h at 37°C. Finally, a microplate reader (Thermo Scientific, Waltham, MA, USA) was employed for the measurement of the optical absorbance at 450 nm.

## Colony formation assay

To detect the effect of let-7b-5p inhibitor and MB-let-7b-5p inhibitor on the proliferative capacity of OVCA cells, colony formation assay was conducted. Transfected SKOV3 and CAOV3 cells were seeded into 6-well plates (5 × 10^2^ cells/well) and cultured for 14 days at 37°C. Every 4 days, the culture medium was replaced with fresh medium. After 14 days of incubation, cells were washed with PBS. Next, at room temperature, cells were fixed with 4% paraformaldehyde for 15 min and stained with 0.5% crystal violet (Sigma-Aldrich; Merck KGaA) for 10 min. The number of colonies containing more than 50 cells was counted and observed under an optical microscope.

## Western blot

Protein levels were detected by western blot analysis. In brief, Radio Immunoprecipitation Assay lysis buffer (Millipore, Merck KGaA) was utilized for the extraction of total protein from cell lines. The concentration of total protein was detected using a BCA Protein assay kit (Nanjing KeyGen Biotech Co., Ltd) following the manufacturer’s guideline. Next, equal amounts of protein (approximately 30 µg) were separated using 12% sodium dodecyl sulfate polyacrylamide gel electrophoresis and then transferred onto polyvinylidene fluoride membranes (Millipore). Next, the membranes were blocked with 5% skimmed milk for 2 h at room temperature and then incubated with primary antibodies overnight at 4°C as below: anti-OCT4 (1/1000; ab181557), anti-Nanog (1/2000; ab109250), anti-SOX2 (1/2000; ab92494), anti-GAPDH (1/10,000; ab181602), anti-DDX19A (1/1000; ab235531). After the primary antibody incubation, the membranes were incubated with horseradish peroxidase-conjugated goat anti-rabbit IgG H&L (1/2000; ab205718) at room temperature for 40 min. The visualization of protein bands was performed by an Enhanced Chemiluminescence Plus kit (EMD Millipore) and the quantification of protein bands was performed by densitometric analysis of protein signals using the ImageJ version 1.49 (National Institutes of Health).

## Sphere formation assay

SKOV3 and CAOV3 cells by different treatments (NC inhibitor, let-7b-5p inhibitor and MB-let-7b-5p inhibitor) were plated on ultra-low attachment 6-well plates (5 × 10^3^ cells/well; Corning Inc., Corning, NY). The cells were cultured in DMEF/F12 (1:1) supplemented with B-27 (Thermo Fisher Scientific), 10 ng/mL epidermal growth factor (EGF, Invitrogen) and 10 ng/mL fibroblast growth factor-basic (bFGF, Invitrogen). After incubation for 10 days, an inverted microscope was utilized for calculating the number of 3-D spheroids containing more than 20 cells.

## Luciferase reporter assay

The binding between let-7b-5p and DDX19A 3ʹUTR was confirmed by the luciferase reporter assay. This assay was performed using the Dual-luciferase Reporter Assay System (Promega, Madison, WI, USA). The wild type (Wt) 3ʹUTR segments of DDX19A at a length of 3100 bp, which were predicted to bind with let-7b-5p by starBase (http://starbase.sysu.edu.cn/index.php), were amplified and inserted into the pmirGLO plasmid (Promega) to construct the pmirGLO-DDX19A 3ʹUTR-Wt vector. The mutant (Mut) DDX19A 3ʹUTR was generated (‘CUACCUC’ sequence was mutated as ‘ACUUUCU’) by a QuikChang Site-Directed Mutagenesis Kit (Agilent Technologies, Santa Clara, CA) and was inserted into the pmirGLO plasmid to construct the pmirGLO-DDX19A 3ʹUTR-Mut vector. The pmirGLO-DDX19A 3ʹUTR-Wt or pmirGLO-DDX19A 3ʹUTR-Mut plasmids were cotransfected with NC inhibitor, let-7b-5p inhibitor, and MB-let-7b-5p inhibitor into SKOV3 and CAOV3 cells using Lipofectamine 3000 (Invitrogen). After transfection for 48 h, Dual-luciferase Reporter Assay System (Promega) was used to assess the relative luciferase activity.

## Statistical analysis

GraphPad Prism was utilized to analyze all the data involved in this study, and data were expressed as the mean ± standard deviation. Each experiment was performed in triplicate. Difference comparison between two groups was analyzed by Student’s t-test and a one-way analysis of variance was applied for the difference comparison among three or more groups. The expression correlation between let-7b-5p and DDX19A in OVCA tissues was analyzed by Pearson correlation analysis. *P* value less than 0.05 was considered of statistical significance.

## Results

### Let-7b-5p is upregulated in OVCA and transfection efficiency of let-7b-5p inhibitor is promoted by USMB

**Figure 1.**
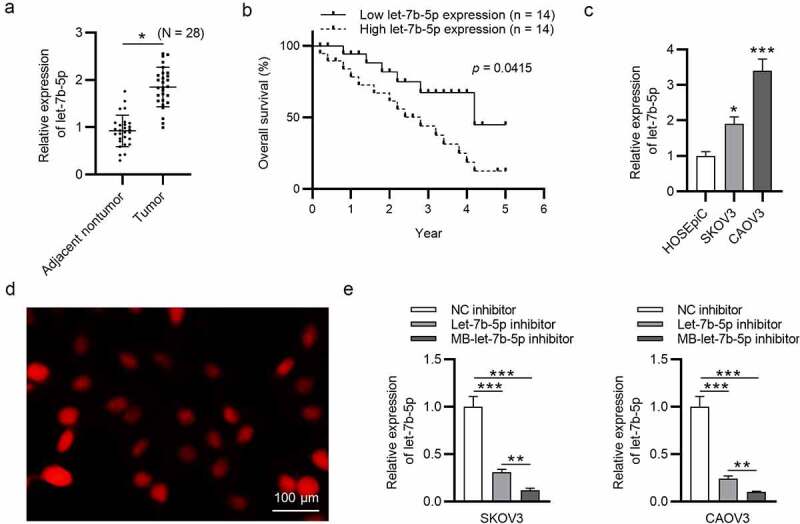
**Let-7b-5p is upregulated in OVCA and transfection efficiency of let-7b-5p inhibitor is promoted by USMB**. (a) Let-7b-5p expression in OVCA tissues (N = 28) was revealed by RT-qPCR analysis. (b) The overall survival of OVCA patients with high or low level of let-7b-5p was presented using the method of Kaplan-Meier analysis. There were 14 patients with high let-7b-5p expression level and 14 patients with low let-7b-5p expression level, which was determined by the average value of let-7b-5p expression in OVCA patients. (c) The level of let-7b-5p in HOSEpiC, SKOV3 and CAOV3 cells was demonstrated by RT-qPCR. (d) The MBs were photographed by a microscope. (e) RT-qPCR was performed to evaluate let-7b-5p expression in SKOV3 and CAOV3 cells after transfection with NC inhibitor, let-7b-5p inhibitor and MB-let-7b-5p inhibitor. *P < 0.05, **P < 0.01, ***P < 0.001

A previous study has mentioned that let-7b-5p is upregulated in OVCA [18], and the upregulation of let-7b-5p expression in our collected OVCA tumor tissues (N = 28) was confirmed by RT-qPCR analysis (Figure 1a). The high expression of let-7b-5p is related to the low overall survival rate of OVCA patients, indicating that the overexpression of let-7b-5p was associated with poor prognosis of OVCA patients (Figure 1b). The high level of let-7b-5p in OVCA (SKOV3 and CAOV3) cells was verified by RT-qPCR analysis (Figure 1c). We found that MBs were opalescent suspension, and the homogeneous parts were spherical and distributed uniformly (Figure 1d). RT-qPCR was performed to detect let-7b-5p level in OVCA cells after transfection with NC inhibitor, let-7b-5p inhibitor, and MB-let-7b-5p inhibitor, which revealed that let-7b-5p inhibitor significantly knocked down the level of let-7b-5p. MB-let-7b-5p inhibitor caused a higher transfection efficiency than let-7b-5p inhibitor (Figure 1e).

### MB-let-7b-5p inhibitor has more suppressive effects on proliferation and stemness characteristics of OVCA cells than let-7b-5p inhibitor

**Figure 2.**
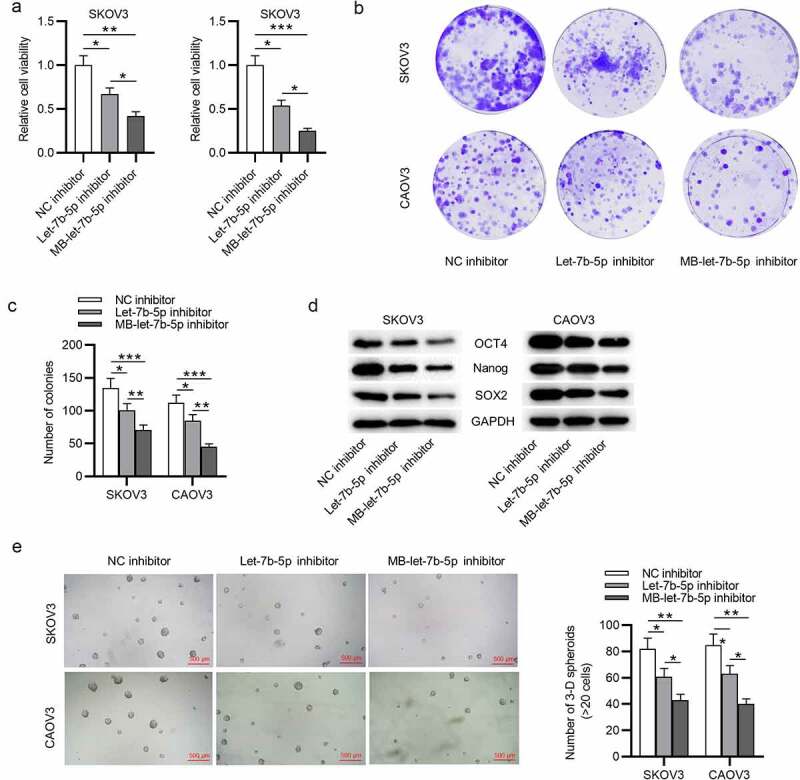
**MB-let-7b-5p inhibitor has more suppressive effects on proliferation and stemness characteristics of OVCA cells than let-7b-5p inhibitor**. SKOV3 and CAOV3 cells were divided into three groups and transfected with NC inhibitor, let-7b-5p inhibitor and MB-let-7b-5p inhibitor. (a) The CCK-8 assay was conducted to evaluate the cell viability. (b-c) Cell proliferative capacity was detected by colony formation assay. (d) Protein levels of stemness markers (OCT4, Nanog and SOX2) in each group were presented by western blot analysis. (e) The number of 3-D spheroids in each group was presented in sphere formation assay. *P < 0.05, **P < 0.01, ***P < 0.001

To further compare the effects of let-7b-5p inhibitor and MB-let-7b-5p inhibitor in OVCA cells, we conducted the following experiments. The CCK-8 assay disclosed that both let-7b-5p inhibitor and MB-let-7b-5p inhibitor inhibited OVCA cell viability. USMB strengthened the inhibitory efficiency of let-7b-5p inhibitor (Figure 2a). Similarly, as presented by colony formation assay, the inhibitory effects of MB-let-7b-5p inhibitor on OVCA cell proliferation were more significant than let-7b-5p inhibitor (Figure 2b-c). Western blot analysis presented that MB-let-7b-5p inhibitor had more inhibitory effects on the protein expression of stemness markers (OCT4, Nanog and SOX2) than let-7b-5p inhibitor (Figure 2d). A sphere formation assay revealed that knockdown of let-7b-5p markedly reduced the number of spheres, while transfection with MB-let-7b-5p inhibitor showed more significant inhibitory effects on the number of spheres (Figure 2e). Moreover, the empty MBs had no significant effects on OVCA cells, and MB-let-7b-5p inhibitor suppressed proliferation and stemness characteristics of OVCA cells (Supplementary Figure 1a-e). To sum up, the inhibitory effects of MB-let-7b-5p inhibitor on proliferation and stemness characteristics of OVCA cells are more significant than let-7b-5p inhibitor.

## DDX19A is downregulated in OVCA

**Figure 3.**
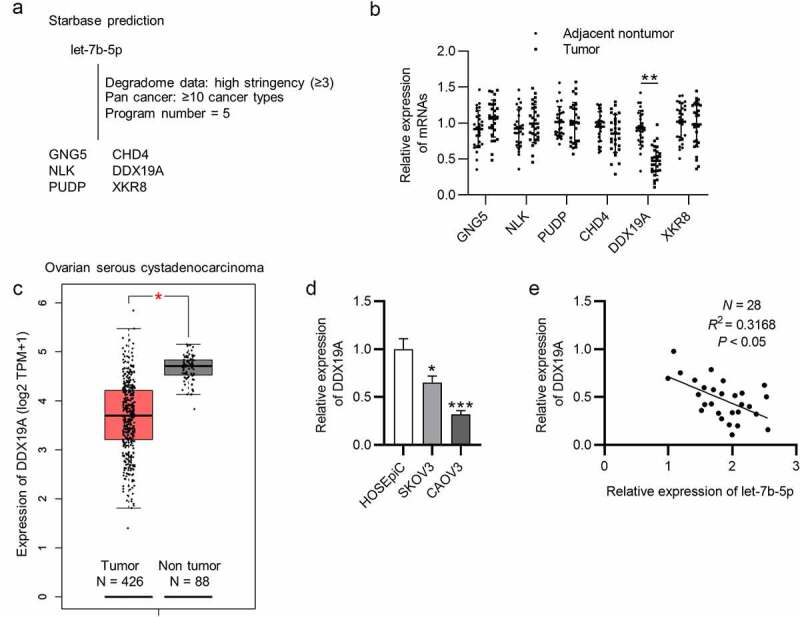
**DDX19A is downregulated in OVCA**. (a) The predicted mRNAs (GNG5, CHD4, NLK, DDX19A, PUDP and XKR8) targeted by let-7b-5p were provided by starBase (http://starbase.sysu.edu.cn/index.php) (filter criteria: Degradome Data: high stringency ≥ 3; Pan cancer: ≥ 10 cancer types; Program number = 5). (b) RT-qPCR was performed to detect the expression of GNG5, CHD4, NLK, DDX19A, PUDP and XKR8 in OVCA tissues (N = 28). (c) GEPIA predicted the expression of DDX19A in ovarian serous cystadenocarcinoma tissues (N = 426) and normal tissues (N = 88). (d) DDX19A level in SKOV3 and CAOV3 cells was measured by RT-qPCR. (e) Pearson correlation analysis presented the correlation between the expression of let-7b-5p and the level of DDX19A in OVCA tissues (N = 28). *P < 0.05, **P < 0.01, ***P < 0.001

The targets of let-7b-5p were searched. We obtained from starBase database (http://starbase.sysu.edu.cn/index.php) the mRNAs that were predicted to be targeted by let-7b-5p, which were GNG5, CHD4, NLK, DDX19A, PUDP and XKR8 (filter criteria: Degradome Data: high stringency ≥3; Pan cancer: ≥10 cancer types; Program number = 5) (Figure 3a). Next, RT-qPCR was performed to assess the expression of these mRNAs in OVCA tissues (N = 28), and it was revealed that DDX19A was expressed at a significantly low level in OVCA tumor tissues (Figure 3b). In addition, we searched GEPIA (http://gepia.cancer-pku.cn/) database and found that DDX19A was downregulated in ovarian serous cystadenocarcinoma tissues (N = 426), which is a phenotype of OVCA (Figure 3c). Subsequently, we conducted RT-qPCR analysis to detect DDX19A expression in SKOV3 and CAOV3 cells. As presented by RT-qPCR, we discovered that DDX19A expression was downregulated in SKOV3 and CAOV3 cells (Figure 3d). To investigate whether DDX19A expression was correlated with the level of let-7b-5p, Pearson correlation analysis was performed to reveal the expression correlation between let-7b-5p and DDX19A in OVCA tissues (N = 28). There was a weak (*R*^2^ = 0.3168) negative expression correlation between let-7b-5p and DDX19A (Figure 3e). Collectively, DDX19A is expressed at a low level in OVCA, and its expression is negatively correlated with let-7b-5p expression.

## Let-7b-5p targets DDX19A 3ʹUTR and suppresses its expression

**Figure 4.**
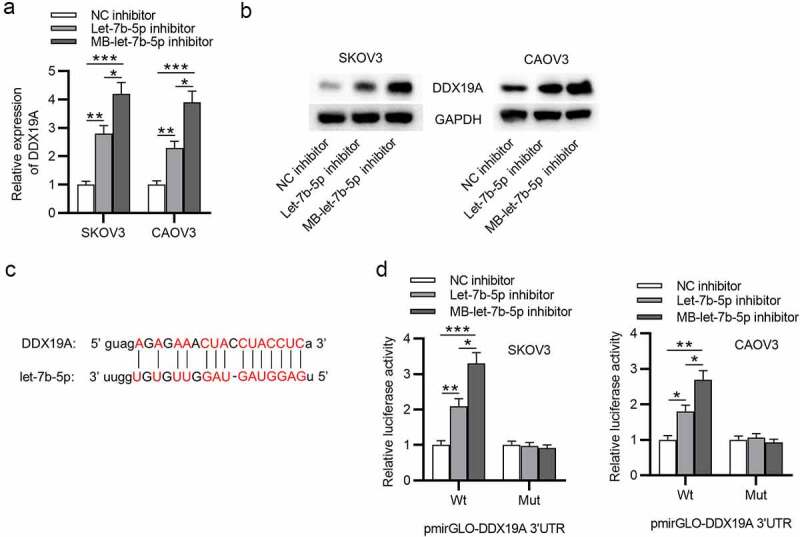
**Let-7b-5p targets DDX19A 3ʹUTR and suppresses its expression**. (a) DDX19A expression in OVCA cells after transfection with let-7b-5p inhibitor and MB-let-7b-5p inhibitor was presented by RT-qPCR. (b) Western blot presented the protein level of DDX19A in OVCA cells transfected with let-7b-5p inhibitor and MB-let-7b-5p inhibitor. (c) The binding sites between DDX19A 3ʹUTR and let-7b-5p were predicted by starBase. (d) Luciferase reporter assay presented the relative luciferase activity of pmirGLO-DDX19A 3ʹUTR after the transfection of let-7b-5p inhibitor and MB-let-7b-5p inhibitor. *P < 0.05, **P < 0.01, ***P < 0.001

The regulatory effect of let-7b-5p on DDX19A was explored. As presented by RT-qPCR, the expression of DDX19A was significantly upregulated after the knockdown of let-7b-5p, and MB-let-7b-5p inhibitor caused a higher increase in DDX19A mRNA expression than let-7b-5p inhibitor (Figure 4a). Consistent with RT-qPCR, western blot analysis demonstrated that compared with let-7b-5p inhibitor, MB-let-7b-5p inhibitor had a more suppressive effect on DDX19A protein (Figure 4b). To testify the interaction between let-7b-5p and DDX19A, we obtained the predicted binding sites of them from starBase database (Figure 4c). A luciferase reporter assay confirmed the binding between DDX19A 3ʹUTR and let-7b-5p and indicated that the lower level of let-7b-5p resulted in higher DDX19A level (Figure 4d). In conclusion, let-7b-5p binds to DDX19A 3ʹUTR and inhibits its expression.

## Silencing of DDX19A counteracts the effects of MB-let-7b-5p inhibitor on OVCA cells

**Figure 5.**
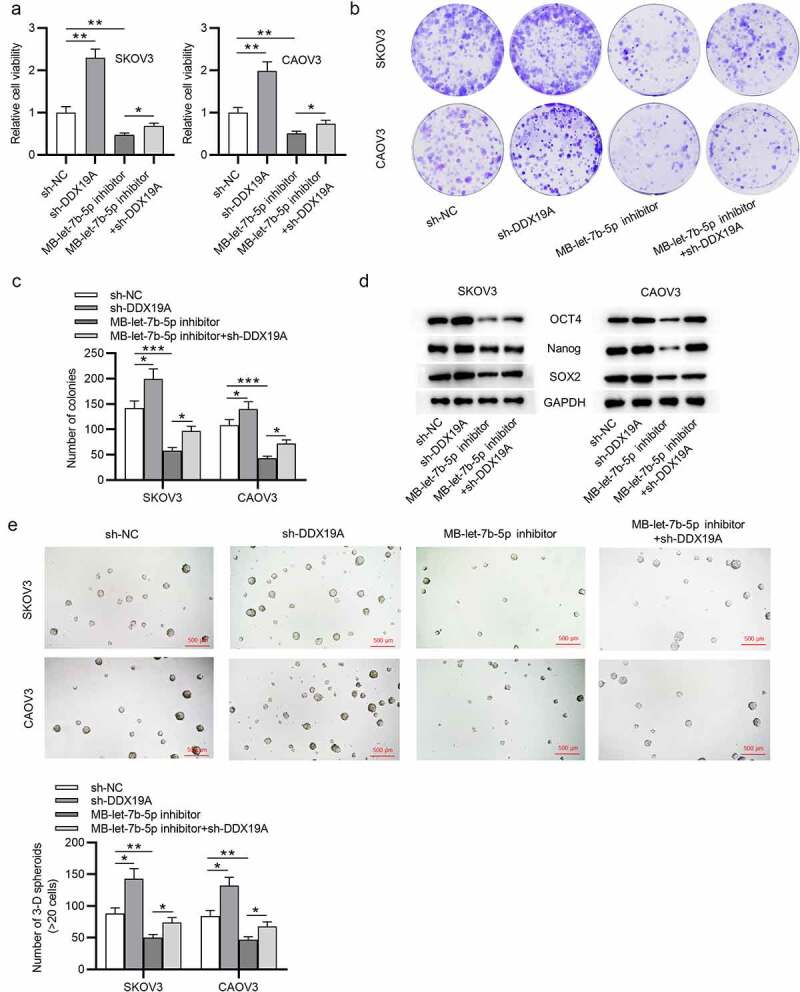
**Silencing of DDX19A counteracts the effects of MB-let-7b-5p inhibitor on OVCA cells**. SKOV3 and CAOV3 cells were divided into 4 groups: sh-NC, sh-DDX19A, MB-let-7b-5p inhibitor, MB-7b-5p inhibitor + sh-DDX19A. (a) Relative viability of cells in the 4 groups was assessed by the CCK-8 assay. (b-c) Colony formation assay was performed to evaluate the proliferation of OVCA cells. (d) The protein levels of stemness markers (OCT4, Nanog and SOX2) were measured by western blot analysis. (e) The number of 3-D spheroids formed by cells in the 4 groups was revealed by sphere formation assay. *P < 0.05

To further explore whether DDX19A is involved in let-7b-5p-mediated cellular processes of OVCA cells, rescue assays were conducted. DDX19A expression was knocked down by transfecting sh-DDX19A into OVCA cells. The CCK-8 assay revealed that DDX19A knockdown promoted cell viability. MB-let-7b-5p inhibition-induced suppression on cell viability was reversed by the silencing of DDX19A (Figure 5a). As presented by colony formation assay, colony formation ability of OVCA cells was promoted by DDX19A knockdown. The inhibitory effect of MB-let-7b-5p inhibitor on the proliferation of OVCA cells was reversed by the transfection of sh-DDX19A (Figure 5b-c). Moreover, western blot demonstrated that inhibition of DDX19A increased protein levels of stemness markers (OCT4, Nanog and SOX2). The suppressive effects of MB-let-7b-5p inhibition on the expression of OCT4, Nanog and SOX2 were partially reversed by the inhibition of DDX19A (Figure 5d). Furthermore, as revealed by the sphere formation assay, the number of 3-D spheroids was reduced by MB-let-7b-5p inhibitor and increased by the transfection of sh-DDX19A (Figure 5e). In summary, knockdown of DDX19A reverses the inhibitory effects of MB-let-7b-5p inhibitor on the viability, proliferation and stemness characteristics of OVCA cells.

## Discussion

According to the results from cancer statistics, ovarian cancer has the highest mortality rate among female reproductive diseases [31]. A common therapy for OVCA is targeting OVCA cells. However, due to tumor recurrence, drug resistance or metastasis, the common therapeutic methods result in poor prognosis [32]. In recent years, extensive studies have revealed that the poor outcomes are associated with CSCs, which are always associated with ovarian cancer recurrence, development, metastasis and drug resistance and are featured with infinite proliferation, self-renewal and multidirectional differentiation in OVCA cells [33]. Therefore, an effective and novel therapeutic target for OVCA aimed at OCSCs is in urgent need. MiRNAs participate in the progression of OVCA, including proliferation, metastasis and EMT [34]. Take miR-585-3p as an example, it was found to suppress the proliferation and migration by directly targeting calpain 9 in high grade serous OVCA [35]. Zhang S *et al*. suggested that miR-193a-5p silencing represses the proliferation and migration and induces apoptosis of OVCA cells via targeting RB binding protein 6, ubiquitin ligase [36].

Preclinical studies on drug and gene delivery to solid tumors have already been applied. Based on these facts, we discussed the potential mechanism of MB-let-7b-5p in the malignant episodes of OVCA. As expected, our results revealed that MB-let-7b-5p inhibitor further strengthened the suppressive role of let-7b-5p inhibitor in OVCA malignancy by targeting DDX19A. The same mechanism of MB-miRNAs on miRNAs has been found in various malignancies. For example, Xiao X *et al*. found that miR-940 suppresses the proliferation of cervical cancer cells and USMB-miR-940 showed the stronger suppressive effects on cell proliferation [37]. The aberrant expression of miRNAs is recognized as the potential biomarkers in different tumors, including OVCA [38]. Although there is a study that mentioned the overexpression of let-7b-5p in OVCA, the exact involvement of let-7b-5p and the promoting effects of USMB treatment on the transfection efficiency of let-7b-5p inhibitor were first studied in this study.

In this study, we observed that let-7b-5p was overexpressed in OVCA and its silencing suppressed the viability, proliferation and sphere formation ability of OVCA cells. Additionally, let-7b-5p silencing suppressed the expression of stemness biomarkers. Moreover, MB-let-7b-5p inhibitor enhanced the inhibitory effects caused by let-7b-5p inhibitor on malignant behaviors of OVCA cells. Let-7b-5p targeted DDX19A 3ʹUTR to degrade DDX19A and further suppress its protein level. The DEAD-box family is characterized by the conserved DEAD motif and is a large group of RNA helicases comprising 37 members [39]. DDX19A (chr16: 70,390,764–70,407,281 at a length of 16,518 bp) is a member of the DEAD-box family. A previous study has shown that DDX19A is overexpressed in cervical squamous cell carcinoma and promotes cell invasion and migration by inducing productions of reactive oxygen species [40]. DDX19A is identified as a prognostic factor of breast cancer disease progression [41]. We found in this study that DDX19A was poorly expressed in OVCA cells, and its expression is negatively correlated with let-7b-5p expression in OVCA tissues. Furthermore, silencing of DDX19A promoted proliferation, stemness characteristics of OVCA cells and reversed the suppressive effects of silenced let-7b-5p on OVCA cells.

However, some limitations of the present study must be stated. First, considering the gap between bench and bedside [42,43], the *in vivo* results were lacking. Efficiency of USMB to enhance gene delivery on subcutaneous tumors and effects of let-7b-5p knockdown on tumor growth in mice will be further explored. Second, the specific mechanisms underlying the antioncogenic role of DDX19A in OVCA remain elusive and deserve further investigation. In addition, it should be noted that the transfection efficiency of miRNA inhibitor using USMB is still low, and further precise effects of USMB should be investigated to improve the gene delivery efficiency.

## Conclusion

Let-7b-5p regulates OVCA cell proliferation and stemness characteristics by targeting DDX19A. USMB increased transfection efficacy of let-7b-5p inhibitor. The USMB-mediated miRNA delivery could be a promising treatment method aimed at OCSCs, providing a novel option for gene therapy of OVCA.

## Supplementary Material

Supplemental Material
