# Supplementary figures and images for: Silencing of let-7b-5p inhibits ovarian cancer cell proliferation and stemness characteristics by Asp-Glu-Ala-Asp-box helicase 19A
Source: Bioengineered. 2021 Oct 6;12(1):7666–77. doi: 10.1080/21655979.2021.1982276 (PMC8806929; doi:10.1080/21655979.2021.1982276)

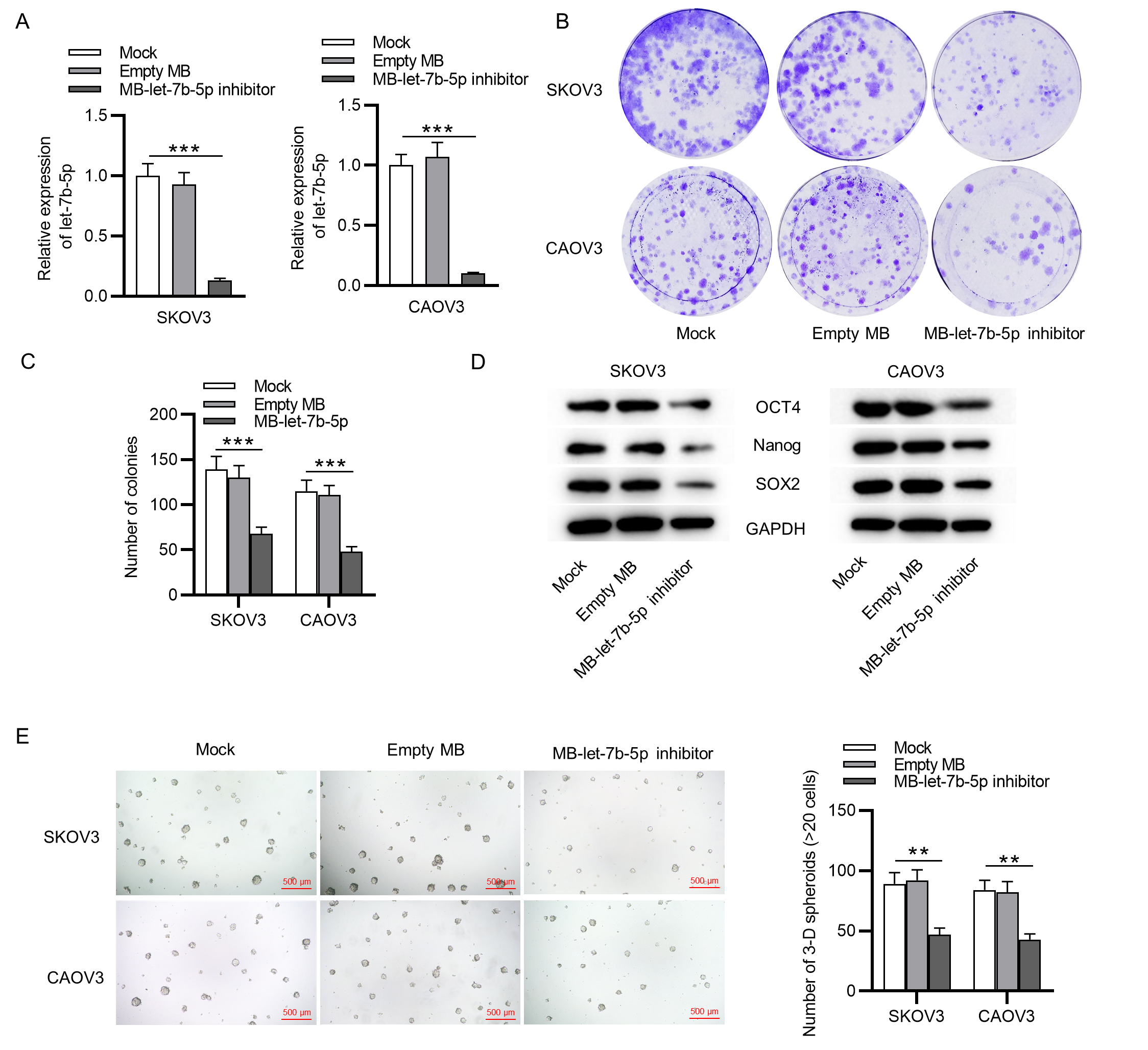

Supplement: Supplemental Material [file KBIE_A_1982276_SM9377.tif]
